# Gains following perceptual learning are closely linked to the initial visual acuity

**DOI:** 10.1038/srep25188

**Published:** 2016-04-28

**Authors:** Oren Yehezkel, Anna Sterkin, Maria Lev, Dennis M. Levi, Uri Polat

**Affiliations:** 1 GlassesOff, Inc. New York, NY, USA; 2 Goldschleger Eye Research Institute, Sackler Faculty of Medicine, Tel Aviv University, Sheba Medical Center, Tel Hashomer 52621, Israel; 3 School of Optometry and Helen Wills Neuroscience Institute, UC Berkeley, Berkeley, CA, USA; 4 School of Optometry and Visual Science, Bar-Ilan University, Ramat-Gan, Israel

## Abstract

The goal of the present study was to evaluate the dependence of perceptual learning gains on initial visual acuity (VA), in a large sample of subjects with a wide range of VAs. A large sample of normally sighted and presbyopic subjects (N = 119; aged 40 to 63) with a wide range of uncorrected near visual acuities (VA, −0.12 to 0.8 LogMAR), underwent perceptual learning. Training consisted of detecting briefly presented Gabor stimuli under spatial and temporal masking conditions. Consistent with previous findings, perceptual learning induced a significant improvement in near VA and reading speed under conditions of limited exposure duration. Our results show that the improvements in VA and reading speed observed following perceptual learning are closely linked to the initial VA, with only a minor fraction of the observed improvement that may be attributed to the additional sessions performed by those with the worse VA.

Presbyopia results from a gradual decrease in accommodative power during normal ageing^1^, leading to reduced near visual acuity and contrast sensitivity^2^,^3^,^4^ and slower visual processing speed^5^,^6^. In addition, visual performance, measured as percent of correct responses for a constant stimulus level or sensitivity (d-prime) is reduced under crowded conditions in the fovea of presbyopic subjects^7^,^8^,^9^. Thus, presbyopia negatively affects the quality of vision for near tasks such as reading. Indeed, near VA declines by about one line (0.1 log unit) on an ETDRS letter chart roughly every five years after the age of 30 years^9^, imposing constraints on near vision even in pre-presbyopic subjects. Because contrast is important for driving neural responses in the visual cortex, blurred input results in weaker and slower neuronal responses in the visual cortex^3^,^10^.

Visual performance on a variety of tasks benefits from practice, in both young^6^,^8^,^11^ and older adults^4^,^8^,^12^,^13^,^14^, resulting in long-term improvements. These long-term effects of repeated practice on a demanding task are termed perceptual learning, for recent reviews, see^15^,^16^,^17^. Visual performance has been shown to improve following practice under crowded conditions, both in normal peripheral vision^18^,^19^,^20^,^21^,^22^ and in the amblyopic fovea^22^,^23^,^24^,^25^,^26^,^27^, for a review see^28^. The specificity of the training effects depends on the amount of training^29^, and the training gains after numerous trials generalize to untrained stimulus parameters, such as untrained spatial separations between stimuli^30^,^31^, for a review, see^32^.

Perceptual learning using a detection paradigm with flankers that facilitate collinear lateral interactions^33^,^34^,^35^,^36^,^37^,^38^ has been shown to improve foveal visual acuity in young normally sighted adults^6^,^8^, and in adults with amblyopia^3^,^25^,^26^,^39^. Similar training, combined with backward masking, resulted in shorter processing times needed for target detection in young normally sighted adults, as indicated by the shorter latency of a specific neuronal marker^40^. A similar training paradigm also resulted in robust improvements in near VA in adults with presbyopia, with no changes in the optics of the eye^4^. Importantly, the gains following training on near-detection-threshold Gabor stimuli generalized to improved performance on high contrast letter stimuli^4^,^6^,^8^.

The goal of the present study was to evaluate the dependency of perceptual learning gains on initial VA, in a large sample of subjects with a wide range of VAs. In uncorrected presbyopia, it is widely acknowledged that the main challenge is in reading small fonts that are beyond the functional reading acuity under the conditions of blur and illumination associated with it. The standard clinical measurements extracted from visual acuity charts reflect acuity for static widely spaced letters. Similarly, the MNREAD chart^41^ provides information relating to the maximal reading speed and its corresponding acuity for font sizes much larger than the effective minimal font size a subject is able to read. Therefore, such standard chart measurements may not be sensitive enough to fully reflect either the real functional performance of a normally sighted adult or the reduction in performance produced by presbyopia. Thus, in addition to the standard measures, we evaluated reading speed with the minimal readable font size on the MNREAD chart.

Consistent with earlier findings^4^,^6^,^8^,^27^, perceptual learning induced a significant improvement in near VA, proportional to the initial VA, with higher gains observed for more advanced presbyopia. Moreover, the transfer of perceptual learning to untrained tasks resulted in improved reading speed, with the gain closely linked to the initial VA as well.

## Results

**Figure 1:**
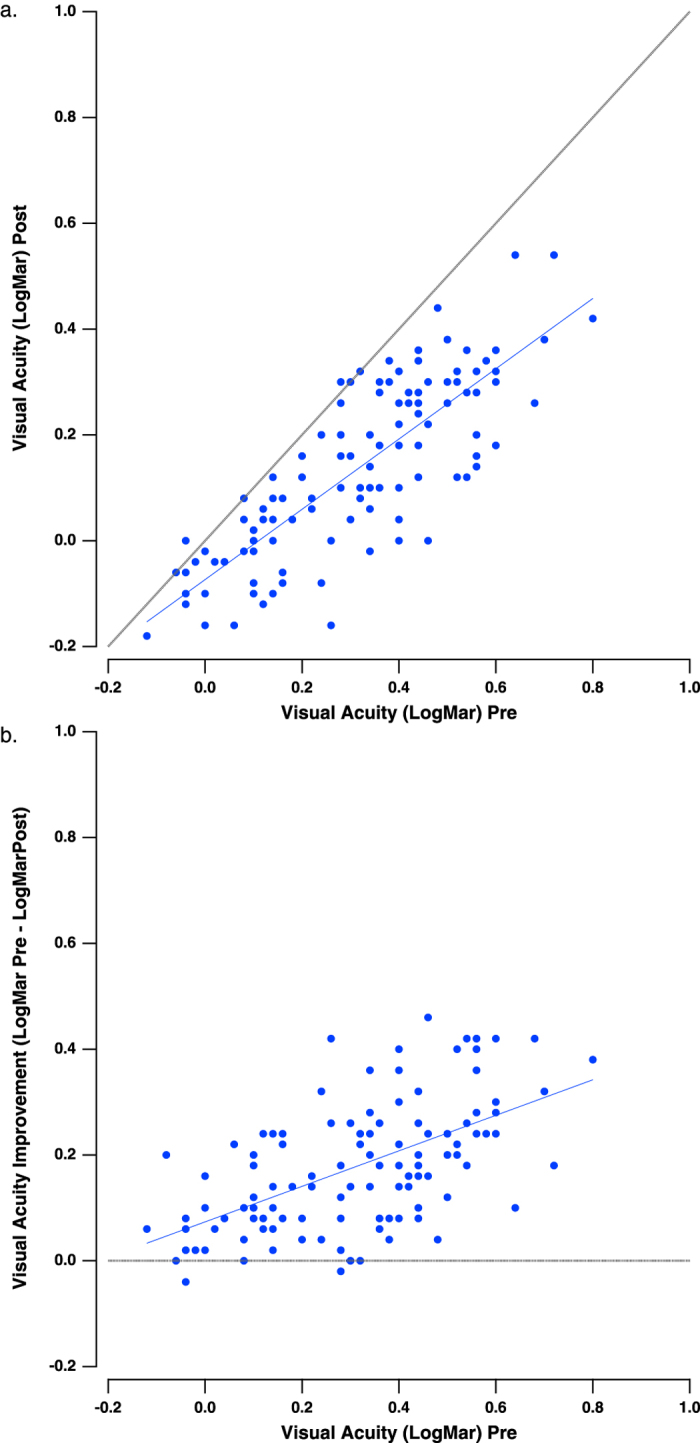
Chart visual acuity. (**a**) Visual acuity (VA) before and after training per subject. The dotted gray line is the unity line, indicating no improvement. The solid blue line is the best fitting regression line. (**b**) The improvement in VA (LogMAR Pre-LogMAR Post) as a function of the initial VA. The dotted gray line indicates no improvement. The solid blue line is the best fitting regression line.

Consistent with our earlier findings^4^,^6^,^8^, there was a significant improvement in VA following training (Fig. 1a,b, the main effect of training (F_1,111_ = 320.5, p < 0.001; VA of 0.313 ± 0.002 at pretest, mean ± se; VA of 0.134 ± 0.001 at posttest).

The improvement in visual acuity was significantly correlated with the initial VA (r = 0.63, p < 0.0001). Subjects with more advanced presbyopia and thus poorer initial VA showed the greatest improvement in VA (Fig. 1b; interaction: group × training, F_7,111_ = 8.7, p < 0.001). This pattern of results shows that improvements after perceptual learning are dependent on the initial acuity^26^,^42^. The main effects of the device type covariate and its interaction with training were both insignificant. The main effects of the session length covariate and its interaction with training were both insignificant.

The improvement in VA was not simply a result of learning to take the test. To evaluate the effect of test-retest reliability, a control group took the pretest and the posttest with no perceptual learning in between (N = 10, age 53 ± 8.75, years old, mean ± SD, ranging between 43 to 68 years old, with VA of 0.17 ± 0.08 for first testing and 0.18 ± 0.08 for the second testing, mean ± se, ranging between −0.12 to 0.58 LogMAR, thus matching the main group in both age range and VA range). This test-retest control group was included in a separate repeated measures ANOVA with a between-subject factor of paradigm (2 levels: with perceptual learning and without). There was a significant effect of perceptual learning as opposed to a simple test-retest effect, evident from the significant interaction between training and paradigm (F_1, 127_ = 23.7, p < 0.001, also see Supplementary information). The effect size measurements indicate there was a large effect of training and its interaction with the initial VA, whereas there was a very small effect of the interaction between training and the device type (partial eta squared values are: 0.31 for the main effect of training, 0.355 for the training × group interaction, and 0.004 for the training × device type interaction and 0.016 for the training × session length interaction). There was a large effect of perceptual learning as opposed to a simple test-retest (partial eta squared value of 0.157 for the training × paradigm interaction).

**Figure 2:**
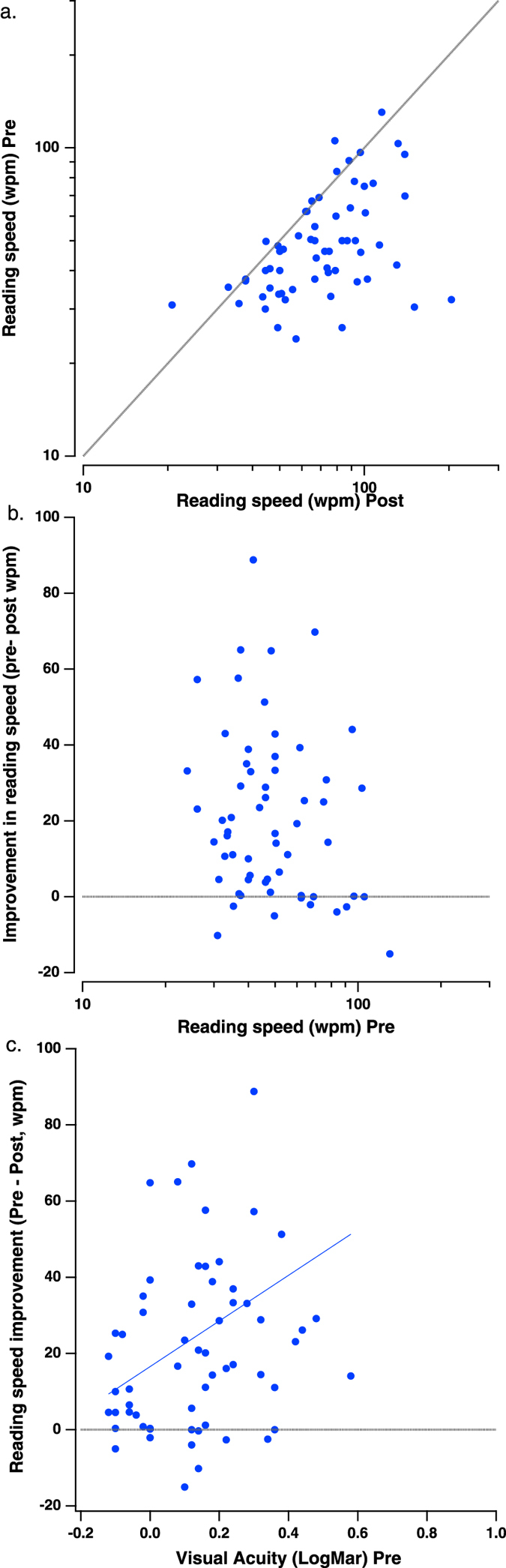
Reading speed. (**a**) Reading speed (wpm) before and after training per subject. The dotted gray line is the unity line, indicating no improvement. Note that pre and post have been switched so that points below the unity line indicate increased reading speed. (**b**) The improvement in reading speed (wpm Pre-wpm Post) as a function of the initial reading speed. The dotted gray line indicates no improvement. (**c**) The improvement in reading speed (wpm Pre-wpm Post) as a function of the initial VA. The dotted gray line indicates no improvement. The solid blue line is the best fitting regression line.

**Figure 3:**
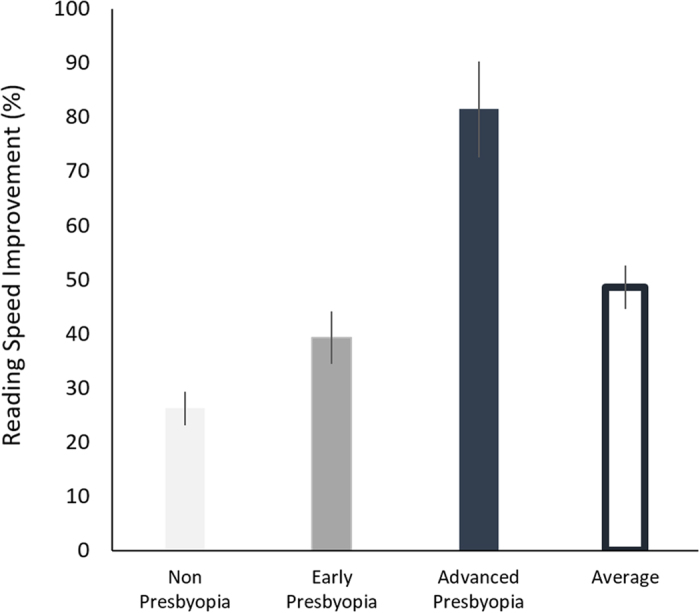
Training gains in reading speed as a function of initial visual acuity. The reading speed improvement, calulated as the difference between the pretest and postest measurements were averaged per 3 levels of initial VA: non-presbyopia, early presbyopia and advanced presbyopia. Errrobars, SE.

A subgroup of 63 subjects (age 48.7 ± 3.4 years old, mean ± SD, ranging between 40 and 58, with initial VA 0.14 ± 0.02 LogMAR, mean ± se, ranging between −0.12 and 0.58 LogMAR) underwent a reading performance measurement before and after training, using an MNREAD chart^41^. Consistent with our earlier findings^4^, there was a significant improvement in the reading performance following the training (Fig. 2). On average, reading speed for the minimal font measured at pretest improved by 50%, from 52 ± 2.8 to 77 ± 4.2 words per minute (wpm), gaining 25 wpm (Fig. 3; F_1,60_ = 40.61, p < 0.001). For the non-presbyopic group specifically (N = 20, initial VA ranging between −0.12 to 0 LogMAR), reading speed improved by 26%, from 53 ± 3.8 to 67 ± 5.8 wpm, gaining 14 wpm, measured for the letter size of 0.02 ± 0.02 LogMAR, mean ± se. For early presbyopia (N = 21, initial VA ranging between 0.06 to 0.18 LogMAR), reading speed improved by 43%, from 55 ± 5.7 to 77 ± 6 wpm, gaining 22 wpm, measured for the letter size of 0.17 ± 0.02 LogMAR, mean ± se. For advanced presbyopia (N = 22, initial VA ranging between 0.2 to 0.58 LogMAR), reading speed improved by 81%, from 47 ± 4.8 to 85 ± 9 wpm, gaining 38 wpm, measured for the letter size of 0.23 ± 0.03 LogMAR, mean ± se. The improvement was also correlated with the initial VA (r = 0.33, p = 0.008), with higher gains observed for those with poorer initial VA (Fig. 2c) and advanced presbyopia levels (Fig. 3; interaction: group × training, F_2,60_ = 3.34, p = 0.042). These results show that, similar to the VA gains, improvement following training in reading speed was also dependent on the initial VA. The poorer the initial VA, the greater the improvement in reading speed.

## Discussion

Consistent with earlier findings^4^,^6^,^8^,^27^, perceptual learning induced a significant improvement in near VA, which was dependent on initial VA, with higher gains observed for those with poorer initial VA and more advanced presbyopia. Similar to the VA gains, improvement following training in reading speed was also correlated with the initial VA.

**Figure 4:**
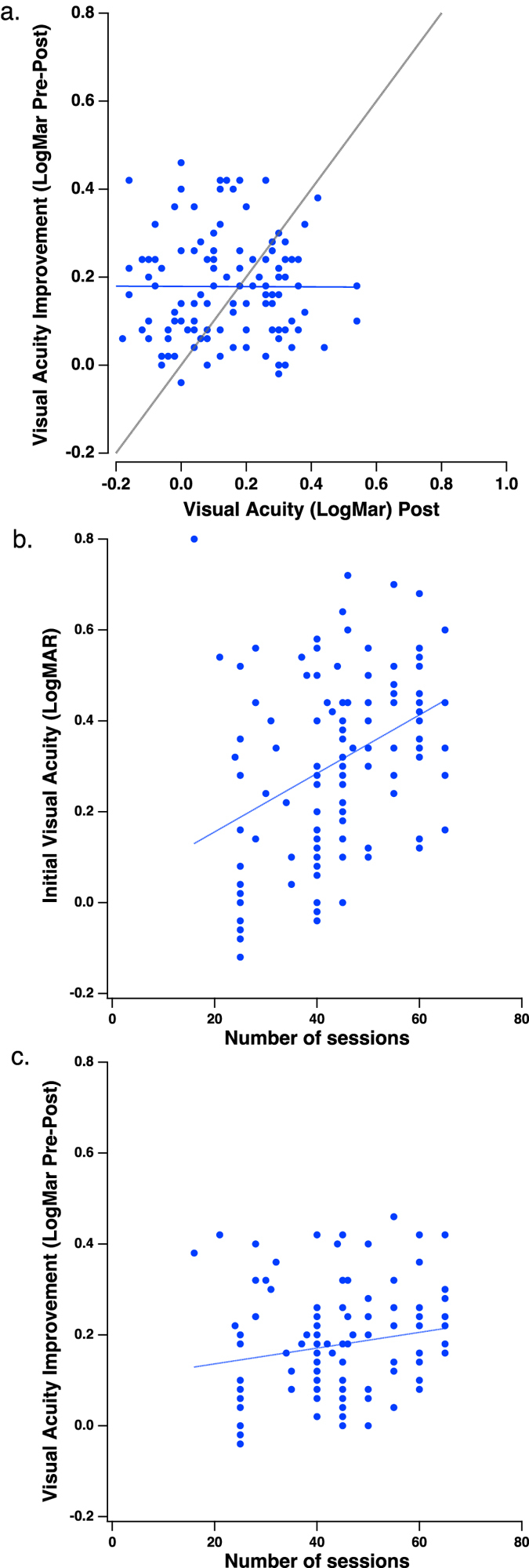
Estimates of VA improvement. (**a**) Improvement in LogMAR (LogMAR Pre-LogMAR Post) vs LogMAR Post. The solid blue line is the best fitting regression line, with a slope of −0.0025. The gray line is the unity line. (**b**) Initial VA as a function of the number of training sessions. The blue line is the best fitting regression line, with a slope of 0.0064. (**c**) VA improvement as a function of the number of training sessions. The blue line is the best fitting regression line, with a slope of 0.0017.

Our results suggest that the improvements following perceptual learning are tied to the initial VA threshold in a lawful way, with higher gains observed for more advanced presbyopia. Interestingly, our data also show very clearly that the improvement is on average 0.18 ± 0.014 LogMAR, independent of the final (post-training) acuity (Fig. 4a). This finding suggests that there may be a fundamental limit (based on optics), and that what is learned is to more efficiently interpret the blurred image - the benefit is that the improvement is a constant fraction (≈a factor of 1.5) of the post training acuity. These results are reminiscent of recent work in normal young observers suggesting that the improvement following perceptual learning is a constant proportion of the initial performance - analogous to Weber’s law^42^. Our results and those of Li, Klein and Levi and of Polat^26^,^43^ in amblyopic subjects suggest that the worse the initial performance, the greater the improvement. However, the Weber’s law behavior suggested by Astle *et al.* implies that the improvement as a function of initial performance should be constant on a log scale, whereas we found that the improvement (in LogMAR units), was not constant – rather the worse the initial performance, the greater the improvement (in LogMAR units). Surprisingly, we find that the improvement (in LogMAR units), is a constant fraction of the post training acuity (Fig. 4a). We suspect that one reason for the discrepancy is that the training in both the current study and that of Li was prolonged, enabling the subjects to reach asymptotic performance, whereas the Astle study used a fixed length of training (10 sessions). Indeed, in our study the algorithm adjusts the training program length automatically according the initial VA and the progress rate, thus the number of training sessions ranged between 25 and 65 and is correlated with the initial VA (r = 0.39, p < 0.001, Fig. 4b).

The fact that those participants with worse initial VA performed more sessions, may suggest the observed greater improvement in these subjects be attributed to the increased number of training sessions *per se*. This may be supported by the study of Li, Klein and Levi^43^ that used “kilo” trials for inducing further improvements. However, here, and as shown by Li and colleagues, the improvement on the training task levels reaches a plateau, which was used as an indication for termination of the training. Thus, we believe that only a minor fraction of the observed improvement (with low marginally significant correlation, r = 0.18, p = 0.05, Fig. 4c) may be attributed to the additional sessions performed by those with the worse VA.

Our results suggest that the gold-standard initial chart VA measurements are useful in assessing the outcome of perceptual learning for near visual acuity and reading speed. On the other hand, static clinical VA may not fully capture the visual losses that occur in presbyopia in a real-life dynamic environment, nor the expected gains in visual performance produced by perceptual learning. Elsewhere we have shown that more efficient temporal and spatial processing following perceptual learning results in faster processing speed and in higher contrast sensitivity^3^,^6^.

The finding that improvements are closely linked to their initial VA threshold in a lawful way might be taken to suggest that the same factors that impose limits on a visual threshold also constrain learning on a visual task^42^. On the other hand, subjects with the poorest performance initially have the most room to improve, while those with the best initial performance may be close to the limit of their performance on the task.

## Methods

We used a previously described structured perceptual learning treatment method for improving visual functions^3^,^4^,^6^,^8^,^40^. Subjects were trained on contrast detection of Gabor targets under backward masking and crowding conditions, imposing both spatial and temporal constraints on the visual processing. The training covered a range of spatial frequencies and orientations that were modified in accordance with the performance improvement (training procedure is described in detail below).

Subjects were trained at a distance of 40 cm with both eyes open. All visual acuity and reading speed measurements were performed before (pre-test) and after (post-test) the treatment. Subjects practiced for at least 3 sessions of about 12–30 min per week over 2 to 4 months. The algorithm adjusts the training program length automatically according the initial VA and the progress rate, thus the number of training sessions ranged from 25 to 65.

### Participants

A total of 119 of normally sighted and presbyopic subjects with no ocular pathology (age 49.28 ± 4.42 years old, mean ± SD, ranging between 40 and 63), with initial uncorrected near VA of 0.313 ± 0.002 LogMAR, mean ± se, ranging between −0.12 and 0.8 LogMAR) volunteered to participate in the study. Ten additional subjects served as controls, (age 52.95 ± 8.75 years old, mean ± SD, ranging between 43 and 68) with initial uncorrected near VA of 0.174 ± 0.07 LogMAR, (mean ± se, ranging between −0.12 and 0.58 LogMAR), participating in pre-and post-testing roughly 2 months apart, but with no intervening training. Thirty of the participants were tested at the University of California, Berkeley, the rest of participants were tested in Israel. Subjects were tested with their habitual distance correction and without any optical correction for near distance. The procedures were approved by the ethics committee of the UC Berkeley IRB and Assuta Medical Center. All participants gave informed written consent to participate in the study. All experimental protocols were performed in accordance with the guidelines provided by the committee approving the experiments.

### Apparatus

Stimuli for the training sessions (Training procedure, see next) were displayed on either a PC (compaq presario CQ62-215DX Notebook) with effective screen resolution of 1366 × 768 pixels at a 60 Hz refresh rate, effective monitor size of 15.6″ (20 × 34 cm) subtending a visual angle of 28.07 × 46.05 degrees, or a mobile iOS device with a color monitor (iPhone and iPod). The effective iOS screen resolution was 640 by 640 pixels (5 cm in diameter), with a pixel size of 0.078 mm (“retina” display, which corresponds to VA of −0.18 LogMAR) and a refresh rate of 60 Hz. The mean display luminance was set to the maximal level (120 cd/m2) in an otherwise dark environment. The Gabor size was maintained constant across devices by appropriate scaling using the device pixel size as a reference.

### Measurements before and after training

A complete eye examination was performed at both pre-test and post-test. The procedures were similar to those of our earlier study^9^. The exam included objective and subjective refraction, ETDRS acuity at a viewing distance of 40 cm, all made while wearing habitual optical correction for far distance and without any optical correction for near distance. All near-acuity measurements were made without any optical correction for the near distance. Standard static near VA was measured using a near ETDRS chart (Precision Vision) from 40 cm, attached to a stand to maintain the exact distance. The ETDRS chart is composed of lines containing 5 letters each with size increments of 0.1 log units and spacing of 1 letter. The measurements were performed in a single session by the same experienced, certified optometrist in the same room under standardized fixed light conditions using 2 variations of the same chart (to prevent participants from memorizing the order of the letters). The termination rules were based on a forced-choice paradigm and testing continued until the participant made a complete line of errors, or read all the letters on the chart^44^. The charts smallest font size is equal to VA of −0.3 LogMAR.

The MNREAD acuity charts are continuous-text reading acuity charts, which are used for subjects with normal and low vision^41^,^45^. These charts, developed for Low-Vision research, can be used to measure reading acuity and speed. The repeatability of MNREAD charts was demonstrated earlier in a study on 30 normal young adults (mean age, 23.3 years), including testing of the variability at different test distances (40 and 52 cm)^46^. The results of the repeated (i.e., test-retest) measurements show repeatability ±0.05 LogMAR for reading acuity and ±8.6 words per minute (wpm) for reading speed. Thus, the MNREAD acuity charts can be used to reliably measure reading acuity, reading speed, and critical print size in a normal adult population. When needed (i.e., for the participants tested in Israel), the MNREAD chart was converted into Hebrew using the original instructions by the author of the MNREAD chart, Prof. G. Legge, maintaining the characteristics of the English version. The task was to read aloud one sentence at a time (black print on white background), as the sentences were uncovered one by one from large to small print. The observers were asked to read as fast and as accurately as possible. Reading time and number of errors made for each sentence were recorded on a score sheet and were converted to reading speed in words per minute by the method described in the test instructions. Viewing distance was fixed at 40 cm.

### Training procedure

The paradigm in this study is similar to that used in our earlier studies involving both young and presbyopic participants^4^,^6^,^8^,^40^ in terms of behavioral tasks and temporal parameters. A perceptual learning method for improving visual functions (using the GlassesOff application) was adapted individually for each user by setting the training parameters according to the user’s performance. Subjects who practiced on PC were trained according to the procedure presented in detail elsewhere^4^, whereas subjects who practiced on iOS devices followed a similar procedure presented in detail in another earlier publication^8^. Participants were trained on contrast detection and discrimination (on mobile devices) of Gabor targets under spatial masking, temporal masking, and spatial crowding conditions, in which spatial and temporal constraints were imposed on the visual processing. The training covered a range of spatial frequencies (2–8 cpd; the size of the Gabor patches ranged from 0.18 to 1 degrees) and included 4 orientations (0, 45, 90, and 135 degrees) that were modified in accordance with the improved performance: every 2 sessions for the orientation and when after reaching a predefined criterion of contrast detection threshold for the spatial frequency. The PC and mobile device screens had an image processing rate of 8 bits precision per color channel (256 unique grayscale tones). Under these specifications the smallest luminance increment at mean luminance is greater than 1% (for typical display gamma values ≥1.8)^47^. We have limited the lowest contrast one could reach to be above 1%, reaching this limit terminated the staircase. Moreover, we were using stimulus composed of Gabor patches and presbyopic subjects, therefore, contrast thresholds received during the training were always higher than this minimal available contrast.

Participants were instructed to train in a dark environment from a distance of 40 cm with both eyes open, while wearing their habitual far distance correction. The algorithm adjusted the stimulus parameters automatically and continuously according the initial VA and the progress rate. Participants performed between 25 and 65 training sessions, on different days, with each session lasting about 12 min (30 min for the subjects in UC Berkeley), with at least 3 sessions per week. At the beginning of training, the number of training sessions was determined by the initial VA, however it was shortened if the performance was better than initially assessed and vice versa. For all participants, training was stopped after performance improvement saturation, however with a minimum of 25 training sessions. The trial number for the participants that used iOS devices varied from ~5250 to 13650 trials (~210 trials per session for 25 to 60 sessions) with an average of ~9240 trials, depending on the individual progress. The trial number for the participants that used PC varied from ~8750 to 21000 trials (350 trials per session) with an average of 13000 trials depending on the personal progress. All of our subjects trained on the same procedure/tasks with two exceptions, the initial target spatial frequency which was determined according to the initial VA and the number of repetitions on some of the tasks that was varied according to the user’s performance. Auditory and visual feedback were provided after each trial. The experimental conditions were as follows: 1) A single Gabor target of a specific spatial frequency; 2) A Gabor target masked by two high-contrast (60%) collinear Gabor flankers with several target-flanker distances; 3) crowding: a Gabor patch masked by multiple (between 4 and 11) collinear or non-collinear iso-oriented flankers, with several target-flanker distances; and 4) temporal masking: backward masking applied the conditions above, composed of either flankers or the crowding condition, presented with varying time intervals (inter-stimulus intervals, ISI) of 68, 83, 116, or 150 msec. The duration of the mask was similar to the target stimulus presentation duration (34, 68, and 116 msec). The ISI, duration that the target and flanking Gabors were presented, their orientation, and spatial frequency were modified between sessions, one parameter at a time, according to the preceding session’s performance.

All measurements and training sessions were conducted without near distance optical corrections. Near visual acuity was measured before and after training. All subjects trained at their homes, with their compliance both monitored online using a back-office client with automatic reminders and notifications and by the study authors.

### Sample Characteristic

The data were tested for normality using the SPSS software (IBM Corp. Released 2013. IBM SPSS Statistics for Windows, Version 22.0. Armonk, NY: IBM Corp). For each of the 16 dependent variables (i.e., per 8 acuity levels in pretest and posttest). A Shapiro-Wilk’s test (P > 0.05)^48^,^49^ and a visual inspection of the normal Q-Q plots showed that the acuity values were approximately normally distributed, for all acuity levels, both at pretest and posttest, with a skewness and kurtosis z-values within the range of ±1.96 (Table 1)^50^,^51^,^52^. Skewness of 0.826 and 0.836 and kurtosis of −0.78 and −0.035 for first and second testing, respectfully.

### Data analysis

VA measurements were divided into 8 levels: bins of 0.1 LogMAR, ranging between below 0 and 0.8 LogMAR. SPSS repeated measures ANOVA with one within subject and one between subjects effect was performed, with a within-subject factor of training (2 levels: before and after perceptual learning) and a between-subject factor of initial VA, denoted as “group” (8 levels). The Huynh-Feldt Epsilon correction was applied. The device type was included as a covariate (3 levels: iPhone, iPod and PC). The session length was included as a covariate (2 levels: 12 and 30 minutes). MNREAD data were divided into 3 groups: non-presbyopia, early presbyopia and advanced presbyopia. We performed SPSS repeated measures ANOVA with one within subject and one between subjects effect, with a within-subject factor of training (2 levels: before and after perceptual learning) and a between-subject factor of initial VA (3 levels). The Huynh-Feldt Epsilon correction was applied.

Pearson correlation with a two-tailed probability values was used for all reported correlations.

## Additional Information

**How to cite this article**: Yehezkel, O. *et al.* Gains following perceptual learning are closely linked to the initial visual acuity. *Sci. Rep.* **6**, 25188; doi: 10.1038/srep25188 (2016).

## Supplementary Material

Supplementary Information

## Figures and Tables

**Table 1:** Data normality testing.

		*VA levels (LogMAR)*							
		up to 0	0–0.1	0.1–0.2	0.2–0.3	0.3–0.4	0.4–0.5	0.5–0.6	0.6–0.8
Pretest	Skewness	−1.87	0.30	0.42	−0.98	−0.27	0.06	−0.07	1.37
	Kurtosis	1.31	−1.40	−0.92	−1.06	−0.85	−0.93	−1.10	0.19
Posttest	Skewness	−1.48	0.10	−0.19	−1.21	0.61	−0.63	−1.06	0.31
	Kurtosis	0.76	−0.28	−1.14	0.10	−1.14	−0.38	−0.90	−0.37

Skewness and kurtosis z-values are presented separately for the 8 acuity levels, at pretest and posttest. All z-values in the table fall within the range of ±1.96.
