# Supplementary material for: Gains following perceptual learning are closely linked to the initial visual acuity
Source: Sci Rep. 2016 Apr 28;6:25188. doi: 10.1038/srep25188 (PMC4848560; doi:10.1038/srep25188)
Supplement: Supplementary Information [file srep25188-s1.pdf]

Gains following perceptual learning are closely linked to the initial Visual Acuity.

Oren Yehezkel[1]*, Anna Sterkin[1,2]*, Maria Lev[2]*, Dennis M. Levi[3] and Uri Polat[1,2]

Supplementary information

The VA data are plotted with the 95% confidence intervals (CIs, Supplementary information Fig. 1) for 3 subgroups of subjects from the trained group compared to the control test-retest group with the matching 3 initial VA levels into which we have binned the data of the 10 control subjects: below 0 LogMAR (4 subjects; panel a), 0.1-0.22 LogMAR (3 subjects; panel b), 0.4-0.6 LogMAR (3 subjects, panel c). The plots show that the training effects are beyond the CI range.

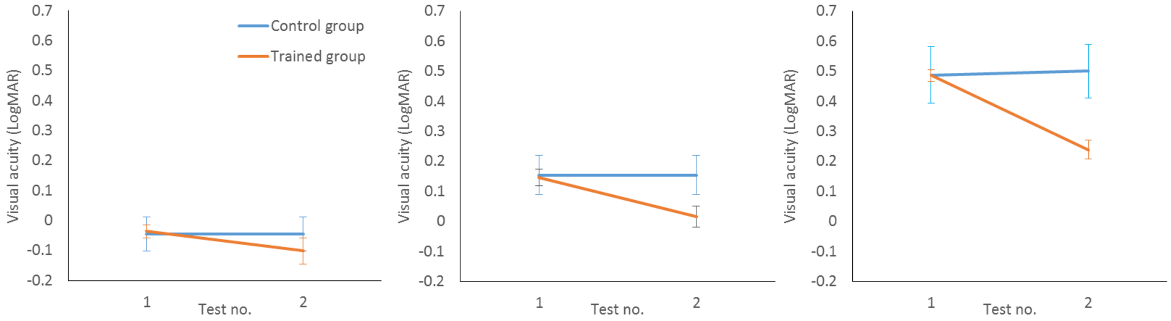

Supplementary information Figure 1. The chart VA data with the 95% confidence intervals (CIs), for the trained (orange) and control (blue) groups, for the 1st and the 2nd testing. Error bars, 95% CIs.
